# Contraception, HIV Services, and PrEP in South African Hair Salons: A Qualitative Study of Owner, Stylist, and Client Perspectives

**DOI:** 10.1007/s10900-019-00698-7

**Published:** 2019-07-06

**Authors:** Ingrid V. Bassett, Sabina Govere, Lucia Millham, Simone C. Frank, Nosipho Dladla, Hilary Thulare, Christina Psaros

**Affiliations:** 1 Division of Infectious Diseases, Massachusetts General Hospital, 100 Cambridge St, 16th Floor, Boston, MA 02114 USA; 2 Medical Practice Evaluation Center, Massachusetts General Hospital, Boston, MA USA; 3 Harvard Medical School, Boston, MA USA; 4 Harvard University Center for AIDS Research, Harvard University, Boston, MA USA; 5 AIDS Healthcare Foundation, Durban, South Africa; 6 Behavioral Medicine Program, Department of Psychiatry, Massachusetts General Hospital, Boston, MA USA

**Keywords:** South Africa, Contraception, HIV prevention, Young women, Hair salon

## Abstract

Women experience challenges engaging with the healthcare system, but frequently utilize hair salons; these are promising venues for family planning and HIV prevention services. Our objective was to assess the acceptability of nurse-offered contraceptive and PrEP services at hair salons in Durban, South Africa. We interviewed salon owners (N = 10) and clients (N = 42) and conducted focus groups with hair stylists (N = 43 stylists; 6 focus groups across five hair salons) to explore barriers and facilitators to providing contraception and PrEP in salons. After developing a codebook, we performed content analysis to identify themes within each conceptual area; 10% of transcripts were coded by two coders to ensure reliability. Content was analyzed according to the following categories: (1) facilitators of and (2) barriers to utilizing these services, and (3) factors to consider for program implementation. Participants identified convenience and female-oriented, supportive atmosphere as facilitators to offering HIV and contraceptive services in salons. Owners and stylists noted that establishing legitimacy was important for program success, including providing promotional pamphlets and employing nurses. Clients cited privacy concerns surrounding HIV testing in a public space as a significant barrier to using these services. Overall, participants were enthusiastic about the program. Convenience and a conducive environment were noted as facilitators to receiving health services in the hair salon; attention will have to be directed to establishing privacy and program legitimacy. Hair salons represent an innovative venue for reaching young women at high-risk for unintended pregnancy and HIV infection.

## Introduction

South Africa has the largest population of individuals living with HIV in the world, with 7.2 million people infected as of 2017, 58% of whom are women [1]. Young women in South Africa are disproportionately affected both by the HIV epidemic and by a high burden of unintended pregnancies. The current annual HIV incidence rate for African women aged 20–34 is 4.5% in South Africa, meaning that four out of every 10 women who are 20 years-old today will have HIV by the time they are 34 [2]. Furthermore, unintended pregnancies account for one-third of all births in sub-Saharan Africa, nearly half of which occur among women age 15–24.

Risk for HIV and for unintended pregnancy is driven by underlying social and structural barriers including poverty, gender inequality, and a lack of autonomous and consistent healthcare access [3]. Compared to older women, young women are more likely to both discontinue contraception and use HIV pre-exposure prophylaxis (PrEP) inconsistently [4–6]. Moreover, the number of young people aged 15–24 in South Africa is expected to triple by 2030 [7]. Thus, in order to stem the tide of HIV among young African women, increased focus on primary HIV prevention and family planning efforts is needed.

The “She Conquers” campaign in South Africa focuses on reducing new HIV infections and unwanted pregnancies among young women [8]. The most recent national family planning policy calls for use of non-clinical settings for contraception provision and for incorporating HIV testing into contraceptive services, but implementation has been limited [9]. Expanding access to contraception and PrEP will require a focus on service delivery systems and user preferences to ensure maximum impact [10].

Hair salons may represent “safe” community spaces where individuals can receive social support. They have been used in the US to promote intimate partner violence screening, but have not been studied for HIV service provision [11]. Given that South African women congregate regularly in community hair salons, these salons could be promising venues for family planning and HIV prevention services. Our objective was to use qualitative methods to assess the acceptability of nurse-provided contraceptive and PrEP services in hair salons in Durban, South Africa.

## Methods

### Study Setting and Participants

We conducted individual qualitative interviews with clients (N = 42) and owners (N = 10), and focus groups with stylists (N = 43) in hair salons in and around Umlazi, an urban township of nearly one million people outside Durban [12–14]. Umlazi is the second most densely-populated township in South Africa with a single hospital in its catchment area [14]. 80% of owners, 93% of stylists, and 100% of clients were female and the average age of each group was 40, 30, and 27 years. Inclusion criteria included: Age ≥ 18 years, English or isiZulu speaking, and able and willing to provide informed consent [15]. Salon owners, stylists, and clients were recruited from a convenient sample of hair salons in Umlazi Township and neighboring communities and were approached by a bilingual (English/isiZulu), female research assistant to assess their interest in participating in an in-depth interview or focus group. Study procedures were approved by the University of KwaZulu-Natal Biomedical Research Ethics Committee (BE388/16) and the Partners (Massachusetts General Hospital/Brigham and Women’s Hospital) Institutional Review Board (Protocol 2016-P-001268, Boston, MA).

### Measures

We used semi-structured interview and focus group guides, developed using guidelines by Huberman and Miles [16] for qualitative data collection. Our questions were informed by domains derived from the Anderson model of health service utilization [17], a review of recent literature on PrEP and contraceptive services, and specific domains believed to be of importance to the study team. Interviews with clients focused on questions about contraceptive use and preferences, knowledge about PrEP, and opinions on offering contraceptive and PrEP services in hair salons. Interviews with hair salon owners focused on topics including roles of hair salons in their communities, programmatic questions about offering these services at hair salons (e.g. feasibility and resources needed, effects on business, and overall comfort level). We probed stylists on a range of topics, focusing on their perceived role of hair stylist in the Umlazi community, their comfort with intervention participation, and useful resources for supporting a health intervention in the salon (e.g. scripts or promotional materials). Questions were open-ended to avoid bias and encourage generation of novel content. Sample questions and probes for each interview and focus group guide are provided in Table 1.

**Table 1:** Sample study content areas and questions/probes

Content area	Sample questions/probes
Clients	
Warm-up questions	• What do you think are the major health care services that young women in Durban need? • Of the services that you mentioned, are there any that you think you would be interested in receiving at the hair salon?
Contraceptive care/family planning	• Could you describe your current contraceptive use (including you current contraceptive method, duration of use, how you chose your current method, your perceived need for contraception, and your interest in contraception)? • What contraceptives are most attractive to you (oral contraceptive pills, injectables, hormonal subdermal implants, intrauterine devices)? • Do you see hair salons as acceptable venues for contraception access and support? Why or why not? • What are your preferences for who to hear reliable information about contraception from at the salon (i.e. hair stylist, peer mentor, nurse)? Why? • Do you think having adherence support for your contraception would be helpful?
HIV testing and PrEP	• What have you heard about PrEP? • How would you feel about HIV counseling and PrEP being offered to clients at the hair salon? • How do you think this would affect salon activities? • What strategies would help you and other clients feel more comfortable and willing to undergo testing at the salon (i.e. park mobile tester right outside the salon, set up private testing area in a back room, etc.)?
Stylists	
Warm-up questions	• How do you think people perceive the role of the hair stylist in the Durban community? • How do you perceive your role as a hair stylist? • How would you describe the relationships you have with your clients?
Programmatic questions	• Do you think discussion health topics and offering services to clients at the salon is feasible? • How do you think this would affect logistics and flow of clients through the salon? • What kind of support might make you feel more comfortable? For example, having a health care provider on site to answer questions
Contraceptive care/family planning	• What kinds of things can make it easy for women to get access to contraception? What kinds of things can make it hard? • What resources might be useful to you as stylists for supporting offering contraception in the salon (i.e. scripts, promotional materials, posters, etc.)? • How could these be implemented? • What do you think about offering some sort of incentive or compensation for offering and accepting contraception at the salon? • Do you think having adherence support for contraception would be helpful?
HIV testing and PrEP	• What have you heard about PrEP? • How would you feel about having HIV testing services offered at the hair salon? • What suggestions would you have about the set-up for offering HIV testing services at the salon?
Owners	
Rapport building questions	• What do you think are the major health care services that young women in Durban need? • How would you describe the role of hair salons in the Umlazi community?
Programmatic questions	• What do you see as potential challenges to discussing health topics with or offering health services for hair salon clients? • Would you feel comfortable having stylists talking with your clients about a health topic? • What resources might be useful for supporting a health intervention in the salon (i.e. scripts, promotional materials, posters, etc.)? Do you have any ideas about how these could be implemented? • What resources do you think you as a salon owner would need if the salon implemented a health intervention? • What are your ideas for these potential incentives or compensation for the salon owners? Stylists? Clients?
Contraceptive care/family planning	• How do you feel about the possibility of offering contraceptive services in the hair salon setting? • Tell me about how you think clients would respond to the possibility of accessing contraception at the hair salon?
HIV testing and PrEP	• What have you heard about PrEP? • How do you feel about the possibility of offering HIV prevention services such as PrEP in the salon setting? • Would you feel comfortable with stylists giving clients information about PrEP?

We paid each participant ZAR 100 for their time. Interviews for client and owners lasted 45–60 min; focus group discussions lasted 1–2 h.

### Analyses

Interviews and focus groups were audio-recorded, transcribed, and translated from isiZulu to English by an independent transcriptionist. Content analyses were conducted to uncover themes related to three category domains: (1) facilitators of and (2) barriers to providing contraception and PrEP in hair salons and (3) program implementation specifics to assess the acceptability of the service and to inform designing future interventions. The analysis was done using an iterative multi-step process. We identified categories and subcategories, and developed a codebook based on those categories. The codebook was organized according to our study question and was aimed at identifying what participants viewed as barriers and facilitators to offering contraceptive services and/or PrEP services in hair salons in Durban, South Africa. Nvivo 12 (2018) was used to code and organize data.

Two coders (SCF and LM) analyzed the first 10% of the transcripts from each group (clients, stylists, and owners) to ensure independent, consistent codebook use. The coders compared results from each phase of their analyses and discussed discrepancies until a resolution was reached. Categories and subcategories outlined in the codebook were continually reexamined to check for applicability and consistency in codebook interpretation. The authors also discussed findings during analysis to ensure that interpretation of the data was not being influenced by perceived theories. An audit trail of coding templates and discussions about the data and computerized coding was kept. Oversight of the qualitative process was provided by CP and the topic-related content was reviewed by IVB and CP.

## Results

Demographic data for clients, stylists, and owners are presented in Table 2. We organized data from 42 client interviews, 6 focus groups with 43 stylists, and 10 owner interviews, into our three category domains. Overall, clients, stylists and owners, as groups, most often reported similar views on common barriers and facilitators to providing contraceptive and PrEP services at hair salons.

**Table 2:** Demographic characteristics

Variable	N	%
A. Clients (N = 42)		
Age, years		
M = 27.1	–	–
SD = 6.3	–	–
Cultural group		
Black (South African)	41	98
Black (Other African)	1	2
Gender		
Female	42	100
Is this salon mostly visited?		
Yes	31	74
No	9	21
Did not answer	2	5
Time spent in salon (hour)		
< 1	6	14
1	20	48
2	11	26
3	5	12
B. Stylists (N = 43)		
Age, years		
M = 29.6	–	–
SD = 5.1	–	–
Cultural group		
Black (South African)	33	77
Black (Other African)	9	21
Did not answer	1	2
Gender		
Female	40	93
Male	3	7
Length of time working at salon (months)		
0–12	13	30
13–24	6	14
25–48	11	26
> 48	12	28
Did not answer	1	2
Works at multiple salons		
Yes	36	84
No	6	14
Did not answer	1	2
Number of working days per week		
5 days/week	1	2
6 days/week	20	47
7 days/week	21	49
Did not answer	1	2
Number of unique clients per week		
0–10 clients/week	6	14
11–25 clients/week	17	40
26–50 clients/week	15	35
> 50 clients/week	3	7
Did not answer	2	4
C. Owners (N = 10)		
Age, y		
M = 40.3	–	–
SD = 7.6	–	–
Cultural group		
Black (South African)	6	60
Black (Other African)	4	40
Gender		
Female	8	80
Male	2	20
Length of time owning salon (years)		
0–5 years	4	40
6–10 years	3	30
> 10 years	3	30
Length of stylist employment		
< 1 year	2	20
1–2 years	3	30
3–5 years	3	30
> 5 years	1	10
Did not answer	1	10
Number of new clients per week		
20–75	6	60
76–150	2	20
> 150	1	10
Did not answer	1	10
Number of salon chairs		
1–5	2	20
6–10	7	70
> 10	1	10
Number of stylists		
1–5	8	80
6–10	–	–
> 10	1	10
Did not answer	1	10
Owner owns multiple salons		
No	8	80
Yes	2	20

*M* mean, *SD* standard deviation

### Facilitators

#### Perceived Value

Participants identified the convenience of being able to receive contraception and PrEP services at the same location and at the same time as their hair appointment as a major advantage to accessing these services in salons. Clients often noted that they prioritize their salon appointments more than they prioritize clinic visits. One 23 year-old (y/o) client explained that her “biggest priority [was her] hair;” another (35 y/o) explained that, “most women try by all means to meet their salon appointments so it would help if we can also receive our contraceptives [at the salon].” A stylist agreed: “most people would rather do their hair than go to the clinics.” One 28 y/o client explained that her salon appointment had so much importance because attending a salon appointment provided visible return on investment. She explains that women understand their beauty, which can be linked with self-esteem and/or self-worth, through the state of their hair: “As working women we hardly get time to go to the clinic but with the salon we don’t take chances. We make it a must that we go to the salon… as women we value our beauty through our hair styles.” A 29 y/o client echoed this sentiment, explaining that while women “can’t go to the clinic and wait for 3 h for their turn … At the salon people know they want to look beautiful, so they end up not minding about the time.” An appointment at a hair salon has tangible value that women can see, an appointment at a clinic feels like long hours for little perceived benefit.

#### Convenience

Additionally, salons are more geographically convenient than clinics and offer more flexible hours. Salons are often located in commercial areas where there are fewer clinics, but which are more convenient to day-to-day activities. One stylist noted that this convenience could favor residents who might not normally be salon customers: “Even if someone is not coming to the salon but works close to the salon, they can come and get their pills from here because they do not have time to go to the clinic.” Additionally, salons are often open on weekends and after business hours to serve clients who work. One stylist noted that “most people are off on weekends and clinics are closed on weekends. So if health services are offered at the salons, the clients would benefit a lot.”

Stylists and owners agreed with clients that offering contraceptive and PrEP services at hair salons would be convenient, adding that they often do not have enough time to go to the clinics themselves because of their work. One 31 y/o female owner explained that “the problem why [*sic*] we hardly go to the clinic is because … it clashes with salon activities;” a stylist felt similarly: “even as an employee of the salon I do not get the time to go to the clinic so I can [get services] from the salon.”

#### Salon Atmosphere

Clients, stylists, and owners also identified the uniquely supportive and peaceful atmosphere found at salons as a facilitator to using these services. Women feel that there is a mutual understanding and support at a salon that may not be present elsewhere. One 25 y/o client explained that, “at the salon even when you are waiting you are with people that you know … [this tends] to make you not worry about the time that you spending [*sic*] at the salon.” In addition, offering PrEP and contraceptives in this environment is not incongruous with topics already discussed at salons. One 20 y/o client explained that “the topics at the salon amongst women always related to [HIV and contraception],” and a 22 y/o client noted that at the salon, “we all have similar interests when it comes to female health issues.”

Many clients, stylists, and owners focused on how the atmosphere at the salon existed because it was a female-dominated space “where women come together,” “feel safe with other women,” and where “the privacy in the salon is between us women. You hardly ever see guys in the salon.” In addition, clients noted a mutual respect for privacy between women that might be lacking in a more heterogeneous environment:

I think I would be comfortable to receive [these services] because there are few men at the salon… if we go to clinics and you see someone who knows you they might think that you are there for HIV. (22 y/o)

In addition, women talked about the mutual understanding that they, as women, have for the health problems that they face that again, might not be present outside of this female-dominated space: “I can get my contraceptives at the salon because we all women … and we all understand that this is a normal thing (28 y/o).

Clients’ focused on salons as good places for women’s health services because of the dearth of men and because as women they understand the importance of these services.

The salon atmosphere was enhanced by salon personnel. Clients and stylists both noted a supportive client-stylist relationship that can foster meaningful conversations. One 28 y/o client explained that she would prefer to get information about contraceptive and PrEP services from her stylist because “we have developed a relationship with the stylist.” The stylists feel similarly, describing clients as friends or family, suggesting that these relationships are not only professional, but personal—built on trust and respect: “My clients are my friends… and my sisters,” “we take them as family,” and “we become counsellors to [clients]…when you are with a client for so many hours, even the problems or situations they have in their lives you can talk to them and advise them.” Overall, the stylists see clients as friends and family; clients divulge personal information and stylists often try to offer advice and assistance on matters outside of hair care.

This supportive and safe atmosphere was contrasted with clinic experiences. A 35 y/o client would rather talk to her stylist because “I am comfortable around my stylist. The nurses are always intimidating and rude.” A stylist also saw this dichotomy, “we would have to be different [than the nurses] … we do everything that the nurses do not do. We have to be kind to the clients and patients.”

One stylist also noted there may be an added challenge to maintaining a comfortable and supportive atmosphere while instituting these services at the salon. The stylist expressed concern that “there are those people who will no longer want to come to [the salon]… some will [talk] negatively about us to other people saying that the salon no longer does hair but now they teaching about HIV.” Given that participants indicated the clinics’ unfavorable atmosphere contributed to their reluctance to go, maintaining an atmosphere at the salon that is different from the clinic is particularly important.

### Barriers

#### Establishing Legitimacy

Clients, stylists, and owners noted that establishing legitimacy is paramount to program success. One 27 y/o client indicated that people are prone to question the legitimacy of the program, “people would think, how do they trust the needles used for injections?.” Participants used words like “joke” or “scam” to describe how the program could be perceived if it was not done in a way that proved to people that it was “genuine,” “serious,” “legit,” or “well thought of.”

Solutions to overcome this barrier included education materials, in the form of posters, pamphlets, and trainings for the stylists. A stylist suggested that posters and pamphlets will “show clients that this is a genuine initiative.” A 42 y/o male owner argued that these materials were necessary for program legitimacy, “If we run the programme without poster or pamphlets, people would think this is a joke or a scam.” A 34 y/o client indicated that “offering a voucher would make people think what you are offering is serious.” Overall, participants suggested that having tangible aspects to the program to “show” people what the program entails would establish legitimacy.

It was also important that clients understood participation was voluntary. One stylist viewed this as a major barrier: “the biggest challenge would be in convincing people…that they are not forced to use this programme.”. Moreover, there may be some hesitation about a program that is offering healthcare services that include prescribing medication outside of a clinic: “people would have to understand that no one is forced to get tested or use the medication” (24 y/o).

A stylist noted that having resources to educate clients about the program would help:

I think there should be trainings and counselling that educates people on the benefits of this programme, this way… they will understand that this programme is aimed at helping them.

Stylists felt they needed training to perform their role in a way that would engender trust in the clients. One 22 y/o client noted that people may be remiss to trust stylists, “a stylist is just a stylist and has no knowledge of health issues.” Clients can often view them as uneducated: “some of the clients have very low regard for us… since we do hair, we are uneducated,” and another, “some people think we just remove dandruff.” Thus, ensuring that stylists are knowledgeable and have proper training is important; one stylist explained, “I think certificates will [assure] clients… that as stylists we know what we are talking about.”

Lastly, while stylists were interested in offering and explaining these services to their clients, participants across groups felt that having a nurse deliver the services would be the best way to promote trust and to maintain some separation between salon and healthcare-oriented activities. One 25 y/o client suggested that, “maybe [the stylists] can just prep us up for it… but when we have to do the actual test it can be the peer advisor or nurse so that your test results can be private and confidential.” Clients explained that there is an assumption that nurses will be “knowledgeable in health-related matters” and have more “experience with health issues,” and that this would garner more trust. One 29 y/o client explained: “People would see it’s a nurse and assume the nurse would do the right thing.” Stylists also mentioned uniforms as a way of establishing legitimacy both for themselves (e.g. caps and T-shirts), and also for the nurses: “if there is someone in uniform… even clients would take the programme seriously.”

#### Privacy Concerns

Clients were most concerned about privacy as a barrier to implementing contraceptive and PrEP services in hair salons, specifically regarding HIV testing. Stigma was at the forefront of participants concerns. Clients explained that they would be worried about someone they know learning private information about their sexual activity because they saw them at the salon:

Some people would just not want to be seen using this service of contraception because they think people would know that they are having sex. (29 y/o)

Imagine you are here to get your prevention pills from the salon and you bump into someone that you know… everyone would know that you [*sic*] getting contraception from the salon. (24 y/o)

Multiple clients also worried that it might be difficult to keep the results of an HIV test private in a salon setting:

If someone gets tested and they find that they are HIV positive and then now come out of the testing room crying… people notice that. (24y/o)

In addition, some clients worried that stylists might gossip. One 25 y/o client explained, “hair stylists are not known for their ability to keep secrets. Stylists are always talking.” Overall, participants worried about losing control of how information about them is shared by using services at a salon where others might know and observe them. These participants were outliers compared to the larger portion of participants who felt that offering services amongst women and supportive stylists would be a positive intervention.

Participants referenced two main solutions to address the issue of privacy in the salon: a private room within the salon or an outside space outside, adjacent to the salon (like a mobile van). Clients who advocated for a private room inside the salon thought it would help ensure utilization:

It would be better if it is inside because people would know while they wait they can go to the nurse and get tested… outside it would take too much time. (29 y/o)

As this participant suggests, offering these services even just outside the salon could be less convenient because it adds an additional step in a different location, which may be problematic because participants saw convenience as a major facilitator of program success. Private rooms do pose a logistical issue; some salons do not have existing spaces that could be used as a private room for this purpose.

Clients who advocated for a mobile van liked the option of going to the mobile van without anyone from the salon observing them:

I would prefer it if there was a mobile van because if I get tested and find out that I am HIV positive you can just put me in the van and give me proper counselling… if they had a private room here in the salon, I would just come out of that room crying so everyone would know what happened. (22 y/o)

Owners also noted that the mobile van would be good solution to address the lack of extra space in many salons. At the same time, a mobile van would be more visible to the public:

If I tell a client to go to the tent and get tested, when the client comes back from the tent I will be able to see if everything is not well…everyone outside will see that this person received bad news. However, if a client tests inside the salon, not many people will see that this person has received bad news. (stylist)

Overall, participants are interested in incorporating this service as discreetly as possible and ensuring privacy for each participant.

### Program Implementation

#### Incentives

Participants felt that incentives would be beneficial to program enrollment, if not necessary, to garner interest among clients. One client noted that incentives have become an expected part of research:

People, patients, or clients expect to get something [from research] because most of the time research has something to offer. (24 y/o)

Others indicated that incentives would help overcome the barrier of legitimacy, “[incentives] would make people think what you are offering is serious” (34 y/o client) and would be an effective way to motivate participants, “free things give better motivations” (24 y/o client).

On the other hand, stylists in particular did not feel that incentives were necessary—clients should want to participate in this program because it is aimed at improving their health and wellbeing. As one stylist explained, “At the end of the day, this is your life, you need to do what is best for yourself, not because there is [*sic*] incentives offered.” A 22 y/o client echoed that she did not think that incentives would help to accomplish the programs goals, “you cannot buy people to take better care of themselves.”

Participants offered a variety of ideas for incentives, beyond money. Many suggested vouchers for airtime or food; others offered ideas more tailored to the program, like sanitary products or hair styling vouchers. Some clients thought that offering sanitary products could both offer something free while simultaneously align with the program’s intentions to promote women’s health—“instead of bring [*sic*] us stuff just to make us happy, you can bring us feminine stuff that would also educate us” (25 y/o). One stylist noted that: “Every girl has a desire to go to the salon but some cannot afford” (24 y/o). Vouchers for salon services of products would help women overcome financial barriers to going to the salon and could simultaneously promote easier access to the health services offered. Owners mentioned that they would want reimbursement for use of the salon space and stylists indicated that they would want extra pay and/or T-shirts and caps to identify themselves as part of the program.

#### Adherence Support

Overall, participants liked the idea of receiving personal SMS messages and having WhatsApp groups as adherence supports. Clients preferred SMS messages for direct adherence motivation because they are more private. One client felt that an SMS could also serve as an automated daily reminder for women on PrEP to take their medication. A few participants also noted that SMS would be more accessible than WhatsApp given data constraints; “WhatsApp will be a problem because I sometimes run out of data. I can receive an SMS even if I do not have data” (35 y/o client).

WhatsApp groups were viewed as a tool that could foster community and provide support. This support could come in the form of “sharing [their] programmes and life experiences” (18 y/o client) or as a place for women to help one another problem solve, “if a participant is having problems with the injection or pills, we can use the WhatsApp group to share ideas on what can be done” (stylist). Overall, participants expressed interest in both personalized SMS reminders and WhatsApp support groups as options for adherence support.

## Discussion

This study explored barriers to and facilitators of offering contraceptive and PrEP services in hair salons in Durban, South Africa. Overall, clients, stylists, and owners were interested in bringing contraceptive and PrEP services to hair salons and believed that it would be possible to successfully implement this intervention. Participants indicated that they saw potential for hair salons to be innovative venues for delivering important healthcare services to women, citing their convenience and supportive, female-dominated environment. Participants did foresee challenges with the program, especially establishing legitimacy to garner trust and ensuring client privacy.

Community interventions can be attractive alternatives to clinic-based care, especially in South Africa where clinics are often overcrowded and inconveniently located. Our proposed intervention aims to use hair salons as “safe” spaces within communities where women can access contraceptive and PrEP services. In the US, hair salons have been successfully used as venues for a variety of health-related interventions. One 2004 study found that clients often discuss sensitive health-related topics with stylists and found that hair salons offered a feasible venue to discuss healthcare matters [11]. A recent study showed the women would disclose experiences of intimate partners violence to stylists in hair salons [18]. Hair salon-based health interventions have yet to be implemented or studied in Sub-Saharan Africa, although our findings suggest that there may be a similar culture around discussing personal topics. A meta-analysis of health promotion and education interventions in hair salons and barbershops in the US found that 73% of them showed significant results [19]. In these interventions, stylists and barbers were often trained to deliver healthcare education to clients, an approach that showed success across health topics (including cancer, hypertension, diabetes, and general wellness). Most of the outcomes, however, were about increased knowledge on health topics, and did not include interventions in which clients participated in an ongoing program or service. However, one barbershop-based intervention aimed at reducing systolic blood pressure in non-Hispanic black men in the United States found that a barber-promoted and pharmacist-led drug therapy led to significantly larger blood pressure reduction than when barbers encouraged patients to make lifestyle modifications and a doctor’s appointment [20]. This suggests that service-oriented interventions in haircare settings can be successful. While service-oriented hair salon interventions have yet to be studied, our research suggests that they are feasible.

A hair salon-based intervention may ameliorate common barriers to PrEP uptake and adherence among young women in South Africa. Women often worry about the stigma associated with taking PrEP. They worry that they might be falsely identified as HIV positive [21] and/or that they will be perceived by others as sexually active [22]. In addition, a dearth of resources and access to reproductive services for women and a lack of social support have added additional barriers to uptake and adherence [22]. Women also cite concerns about PrEP’s side effects as a reason for non-adherence [21]. Through our planned intervention, we seek to address many of these barriers by increasing accessibility to services at a community-level and by offering services in a “safe,” comfortable environment where women receive social support and education on the services provided by people with whom they already have close relationships. Additionally, the female-dominated atmosphere, where participants felt there was acceptance and understanding of the importance of these services, suggests that offering contraception and PrEP in hair salons could be a helpful way to reduce stigma and focus on prevention as part of wellness. Participants worried about the potential side effects of PrEP and indicated that having pamphlets and posters to properly educate clients would help establish program legitimacy and assuage these concerns.

We present a novel and viable approach to address some of the most pressing public health concerns facing women in South Africa through assessing the feasibility of offering PrEP and contraceptive services in hair salons. These qualitative data can directly inform implementation of this intervention. Such an intervention needs to emphasize privacy, convenience and support for participants and be perceived as legitimate and trustworthy. Privacy can be prioritized through creating a separate space for health-related services, while remaining associated with the salon and maintaining the salon environment. Clients were open to both private rooms and mobile vans as potential spaces for services; owners were concerned about space issues associated with private rooms while clients did generally prefer the idea of a mobile van. Posters displayed in salons and pamphlets distributed to interested clients can establish legitimacy and incorporating positive messaging and destigmatizing campaigns into these materials could encourage and maintain an atmosphere distinct from those at clinics. While clients often cited nurses as mean and rude, they were important to involve for legitimacy. It may help to provide additional training to nurses to mitigate this dichotomy. We can also provide training for stylists and maintain a nurse on site to deliver injections, perform HIV testing, and dispense PrEP. Participants felt mixed about offering incentives to program participants, which will need to be considered as something that may impact implementation.

This study should be considered in the context of its strengths and limitations. We sampled participants with a variety of perspectives and roles (clients, stylists, and owners) from multiple different hair salons in Umlazi. We asked open-ended questions in a semi-structured format that allowed participants to explore subjects more in depth if they wished, but also created a baseline level of comparability between participant responses. We did not ask participants to report their HIV status, which may have influenced their views on HIV testing and PrEP services. Questions about willingness and interest in PrEP services may have been influenced by the participants current HIV status. Pre-existing knowledge of PrEP was limited. Therefore, study staff had to explain what PrEP was, and the centrality of HIV testing to PrEP provision. While women under 18 years are at also high risk for unintended pregnancy and HIV and may have their own unique set of barriers and facilitators to hair salon-based services, they were not included in our sample. Despite these limitations, this study conveys an overall willingness of clients to participate in receiving contraceptive and PrEP services in hair salons and eagerness of owners and stylists to offer such services to women in Umlazi. In this qualitative study of hair salon owners, stylists, and clients in Umlazi Township, South Africa, convenience and a conducive environment were noted as facilitators to receiving health services in hair salons. Establishing privacy for HIV testing and program legitimacy through advertising will be paramount. Hair salons represent an innovative venue for reaching young women at high-risk for unintended pregnancy and HIV infection by capitalizing on the focus on convenience and comfort that salons provide.
